# Is Elevation of Alkaline Phosphatase a Predictive Factor of Response to Alectinib in NSCLC?

**DOI:** 10.3390/curroncol29010016

**Published:** 2021-12-31

**Authors:** Walid Shalata, Alexander Yakobson, Rachel Steckbeck, Ashraf Abu Jama, Omar Abu Saleh, Abed Agbarya

**Affiliations:** 1 The Legacy Heritage Oncology Center & Dr. Larry Norton Institute, Soroka Medical Center, Ben Gurion University of the Negev, Beer Sheva 84105, Israel; alexy@clalit.org.il (A.Y.); ashrafag@clalit.org.il (A.A.J.); omarasa@clalit.org.il (O.A.S.); 2 Medical School for International Health, Ben Gurion University of the Negev, Beer Sheva 84105, Israel; steckbec@post.bgu.ac.il; 3 Oncology Department, Bnai Zion Medical Centre, Haifa 31048, Israel; abed.agbarya@b-zion.org.il

**Keywords:** lung adenocarcinoma, ALK mutation, Alecensa^®^ (Alectinib), alkaline phosphatase (ALP), toxicity

## Abstract

In the following report, we describe a case of alkaline phosphatase (ALP) elevation occurring during treatment with alectinib (Alecensa™), which was administered for anaplastic lymphoma kinase (ALK) mutated metastatic non-small cell lung cancer (mNSCLC). A 51 year-old female with widespread metastatic disease exhibited a rapid and significant response within a very short period to alectinib therapy, accompanied by a rapid increase of ALP to more than six times the upper limit of normal (grade 3) ALP, decreasing to within normal limits within 3 weeks after initiation of therapy without any dose modification.

## 1. Introduction

Lung cancer remains the most common cause of cancer-related deaths in the United States and is a significant health care concern throughout the world [1]. NSCLC (non-small cell lung cancer) accounts for over 80% of all lung cancers. NSCLC has an insidious natural history, with few if any symptoms until the illness has spread widely. As a result, the majority of lung carcinomas are discovered at an advanced stage, with a dismal prognosis [2].

Anaplastic lymphoma kinase (ALK) gene rearrangements, which result in the EML4-ALK fusion oncogene, are found in approximately 3–5% of NSCLC advanced disease, [3] mainly in never-smokers or light smokers and particularly in more youthful female patients (median age of 50), with adenocarcinoma where the proportion of ALK rearrangement cases reaches up to 33–30% [4,5].

Alectinib (Alecensa™) is a second-generation, highly selective and potent central nervous system (CNS)-active ALK inhibitor that inhibits tumor proliferation, with specific activity against several ALK mutations [6,7,8]. Alectinib was approved by the US Food and Drug Administration (FDA) and by the European Medicines Agency (EMA) as a first-line treatment for patients with ALK mutations with metastatic NSCLC and for those who progress on crizotinib as a first line treatment, based on the data from the phase 3 ALEX trial [8,9,10]. In that study, alectinib 600 mg orally twice per day was compared to crizotinib 250 mg orally twice per day in patients with ALK mutation positive advanced disease of non-small cell lung cancer, with significantly better progression-free survival at 12 months with alectinib (68.4%) than with crizotinib (48.7%) [8].

Alectinib is associated with a modest elevation in alanine transaminase (ALT), aspartate transaminase (AST) and bilirubin, as well as renal insufficiency during therapy (less than 3% of patients). In rare cases, alectinib may cause elevated alkaline phosphatase (ALP). Among all of the reported side effects that had greater than grade 2 toxicity, elevated ALP accounted for less than 1% of those cases [11,12].

ALP is found on the outer layer of the cell membrane and catalyzes the hydrolysis of organic phosphate esters in the extracellular environment at basic pH values. It has a half-life of 7 days and more than 80% of the ALP in circulation originates from bone, liver and intestine [13,14].

We present, to the best of our knowledge, the first reported case of ALP elevation, with a rapid response of NSCLC in patients treated for ALK-mutated NSCLC. 

## 2. Case

**Figure 1:**
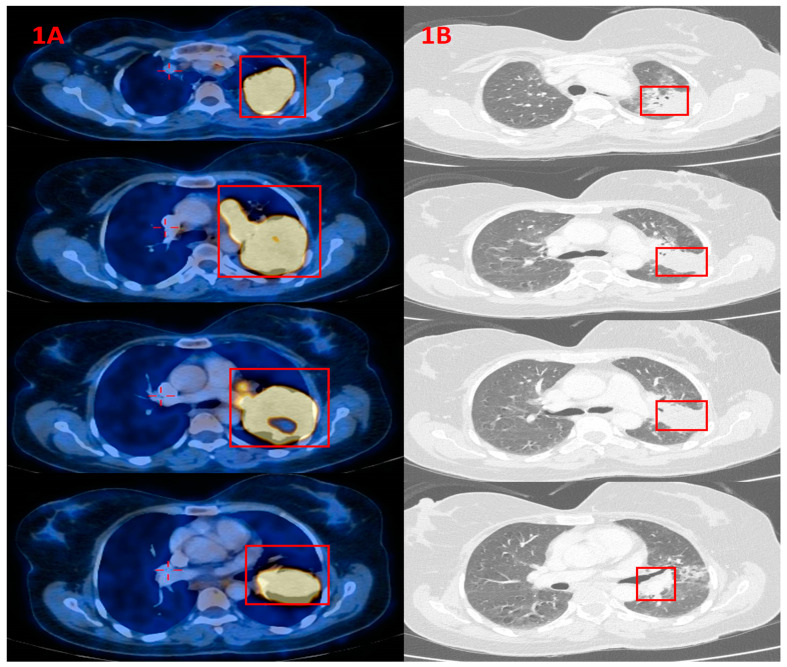
(**A**) PET-CT (29 April 2021) with hypermetabolic uptake of the tumor in the left lung (red squares), before starting alectinib. (**B**). CT scan of chest (12 July 2021) showing radiological significant improvement, with the left lung mass having decreased in diameter (red squares) after starting alectinib, along with elevation and subsequent normalization of ALP values.

In February 2021, A 51-year old female (a housewife) presented with a dry cough and dyspnea of 3 months’ duration, chest discomfort and weight loss (5 kg in the previous 4 months). She was a non-smoker, generally healthy, on no medication and with no family history of cancer. Chest radiography (CXR) showed a ground-glass opacity (GGO) in the left upper lobe (LUL) (diameter 6 cm) and diffuse smaller GGOs in both lungs. Pneumonia was suspected and she was treated with antibiotics. One month later she underwent follow-up CXR, which revealed a LUL and diffuse GGOs in both lungs (the same opacities that were seen a month before). For further investigation, the patient underwent chest computed tomography scan (CT), which showed a LUL mass with a size of 8.7 cm × 4 cm, diffuse GGO in both lungs (the largest 1 cm in diameter) and enlarged mediastinal lymph nodes (bilateral). Bronchoscopy was performed with biopsy of the LUL mass. Pathological results showed adenocarcinoma of lung origin. Magnetic resonance imaging (MRI) of the head showed no evidence of brain metastases. Positron emission tomography–computed tomography (PET-CT) showed hyper-metabolic uptake in the LUL mass (with a size of 9.5 cm × 5 cm) and a mass with hyper-metabolic uptake involving the left sternum area and left hilum which was connected to the mass in the LUL (with a size of 1.8 cm × 5 cm). In addition, hyper-metabolic uptake in the diffuse GGOs in both lungs was noted (the largest was 1 cm in diameter), as well as hyper-metabolic uptake in enlarged right mediastinal and hilar lymph nodes (Figure 1A). Complete blood count and chemistry panel, including ALP, were all within the normal range.

The presumptive clinical diagnosis was stage T4 N2 M1 (stage 4 (metastatic)) NSCLC. Molecular testing was positive for ALK rearrangement. In May 2021, the patient started alectinib treatment (600 mg BID). Three weeks after initiation of therapy, the patient reported improvement of dyspnea and chest discomfort, with reduced cough. Laboratory examination showed complete blood count in the normal range, but blood chemistry showed elevated ALP of 815 U/L (normal range 30–120 U/L), AST 60 U/L (normal range 0–35 U/L), ALT 86 U/L (normal range 0–45 U/L), calcium 7.6 (normal range 8.5–10.5 mg/dL), potassium 5.7 (normal range 3.5–5.0 mg/dL) and urea 53.2 (normal range 17–4.3 mg/dL). Bilirubin was in the normal range. Three weeks later, a blood chemistry test showed ALP 139 U/L, AST 33 U/L and ALT 38 U/L, with no other pathological findings. In July 2021 (less than 2 months after the first dose of alectinib), the patient underwent a chest CT scan for follow-up, which showed significant improvement, with the LUL mass having decreased in size to 3.4 cm × 3.4 cm, the mediastinal and right hilar lymph nodes having decreased to normal size, and the disappearance of the lung nodules (GGOs) (Figure 1B). A blood test during follow-up showed ALP, AST and ALT all within normal ranges.

## 3. Discussion

**Figure 2:**
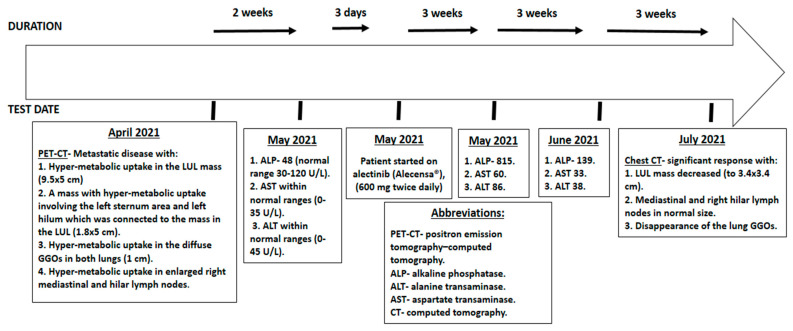
The patient’s time line for cancer treatment and alectinib-induced elevation of ALP.

We have described a patient who had a serious adverse event—ALP elevation to more than six times the upper limit of normal, which in very rare cases has been known to be caused by TKIs (tyrosine kinase inhibitor) during treatment. ALP can be elevated in different situations, including from malignant spread of oncologic disease to liver and bone as well as diseases of the placenta and intestines. Elevation of ALP as an adverse effect of TKIs usually occurs during the first 3 months of treatment. The withholding of treatment in most cases reverses these abnormalities [13,15,16]. Despite being elevated to a severe level, since ALP has been known to rise as a result of alectinib therapy, there was no special recommendation from the manufacturer regarding dose adjustment [16]. Our patient was asked to continue treatment (full dose) with close follow-up. At the 3-week follow-up, the patient had a rapid disease response, including a complete remission of widespread metastatic disease and significant reduction in size of the primary tumor, along with serum ALP returning to the normal range (Figure 2).

The significant and rapid response of the disease raises the question of whether the early occurrence of the exacerbation of the ALP was a TKI-related toxicity (although not reported in previous studies to be elevated to such a level) [8] or whether it was predictive of a dramatic tumor response to TKI-based therapy. It has been shown that in the treatment with new TKIs and immunotherapy that the presence and severity of rare adverse events may be a predictive factor for extraordinary responses [17,18].

The elevation of ALP in this situation could be a result of the tumor tissue breakdown and destruction, a sign of an early response to treatment, and tumor lysis-syndrome (TLS). TLS causes abnormalities in chemistries (hyperkalemia, hyperphosphatemia, hyperuricemia and hypocalcemia), caused by rapid tumor breakdown. TLS is most commonly seen as a result of treatment of lymphoma, leukemia and multiple myeloma [19,20,21]. It has also been shown that in rare cases, ALP may be elevated as a result of tumor tissue breakdown [21]. Furthermore, the elevations of the ALT and AST in this situation could be a result of the toxic tissues that were excreted into the blood—an etiology consistent with the decrease to a normal value while the primary tumor significantly decreased in size, along with the metastatic findings.

## 4. Conclusions

When ALP is elevated, it is essential to rule out causes such as bone metastasis, gallstones, hepatitis, cirrhosis, serious infection and pregnancy. In our case, the clinical setting and appropriate imaging studies excluded these causes. Further investigation is important for understanding the clinical course and therapeutic effects of patients under treatment with alectinib in similar clinical settings.

## Data Availability

The data presented in this study are available on request from the corresponding author.
